# Brittle Cornea Syndrome: Case Report with Novel Mutation in the *PRDM5* Gene and Review of the Literature

**DOI:** 10.1155/2015/637084

**Published:** 2015-06-29

**Authors:** Georgia Avgitidou, Sebastian Siebelmann, Bjoern Bachmann, Juergen Kohlhase, Ludwig M. Heindl, Claus Cursiefen

**Affiliations:** ^1^Department of Ophthalmology, University of Cologne, 50937 Cologne, Germany; ^2^Center for Human Genetics, 79100 Freiburg, Germany

## Abstract

A 3-year-old boy presented with acute corneal hydrops on the left eye and spontaneous corneal rupture on the right eye. A diagnosis of brittle cornea syndrome was confirmed by molecular analysis. A novel mutation, the homozygous variant c.17T>G, p.V6G, was found in the gene for PR-domain-containing protein 5 (PRDM5) in exon 1. Brittle cornea syndrome is a rare connective tissue disease with typical ocular, auditory, musculoskeletal, and cutaneous disorders. Almost all patients suffer from declined vision due to corneal scarring, thinning, and rupture. The most common ophthalmologic findings include keratoconus, progressive central corneal thinning, high myopia, irregular astigmatism, retinal detachment, and high risk for spontaneous corneal or scleral rupture. In addition to describing the case with a novel mutation here we review the current literature on brittle cornea syndrome pathogenesis, clinical findings, and therapy.

## 1. Introduction

Brittle cornea syndrome is a rare autosomal recessive disease with generalized connective tissue damage [1]. Classical ophthalmologic findings include extreme corneal thinning (220–450 *μ*m) [1], irregular corneal astigmatism, high myopia, blue sclera, progressive keratoglobus or keratoconus, and retinal detachment [1–3]. Mutations in the Zinc-Finger-469 (ZNF469) gene are causative for brittle cornea syndrome type 1, and mutations in the gene for PR-domain-containing protein 5 (*PRDM5*) determine brittle cornea syndrome type 2 [1–4]. Corneal thickness is strongly associated with ZNF469 and PRDM5 gene products [1, 3, 5]. It is obscure how mutations in these genes cause typical features of this syndrome, but the gene products, both containing DNA binding zinc finger domains, seem to play a role in the transcriptional regulation of extracellular matrix genes, including corneal fibrillar collagens [5, 6]. Alterations in this pathway lead to changes in corneal integrity due to corneal thinning [5–7]. Homozygous mutations in the genes* ZNF469* or *PRDM5* often result in early and severe keratoconus, corneal thinning, and blue sclera. Heterozygous mutations are usually associated with milder corneal thinning and keratoconus with blue sclera [1]. Due to extreme corneal thinning corneal structural integrity is reduced and corneal fragility often leads to corneal rupture either after minor trauma or spontaneously [2, 3], so that nearly all patients suffer from declining vision due to corneal rupture and scars [1–3]. Since brittle cornea syndrome is part of a generalized connective tissue disease, ZNF469 and PRDM5 gene products concern the development of extracellular matrix [3]. Mutations in these genes are supposed to work like “loss-of-function alleles” [3] affecting fibroblasts [3, 6]. Such mutations in fibroblasts cause different disorders in collagens, integrin, or fibronectin [5], determining extraocular dysfunctions as a generalized connective tissue disorder including auditory, skin, and musculoskeletal features [6].

## 2. Case Report

A 3-year-old boy presented with a whitish-clouded cornea and loss of vision on the left eye for one week and a physiologically appearing right eye. There was no trauma remembered or pain, family history showed no conspicuous eye disorders, no infections, or abnormalities in pregnancy or birth, and no genetic disorders were known. General pediatric physical examination was normal for all systems. The parents had Turkish origin, and history of parental consanguinity is known.

The clinical examination under general anesthesia showed a blue discoloration of sclera in both eyes. Intraocular pressure was within normal levels (8 mmHg in the left eye and 10 mmHg in the right eye). The posterior segment was examined by standardized echography and showed no retinal anomalies or retinal detachment. Axial length was 21.7 mm in the right eye and 22.1 mm in the left eye, suggesting myopic eyes. Retinoscopy in cycloplegia showed −4.0 diopter and astigmatism of −4.5 at 168 degrees on the right side. Retinoscopy in the left eye was not possible.

The cornea of the left eye revealed complete corneal edema with obvious stromal and bullous epithelial keratopathy. The epithelial layer was closed. Central corneal thickness was 745 *μ*m in the left eye and the mean value of thickness peripherally was 550 *μ*m as measured by ultrasound pachymetry (PalmScan AP 2000, Micro Medical Devices, USA).

The right eye showed corneal thinning with a central thickness of 212 *μ*m and a peripheral thickness of 308 *μ*m with corneal astigmatism in topography (−4.5D at 169 degrees). The inferior paracentral cornea showed a deep stromal scar with the remaining corneal surface clear. Funduscopy showed normal optic nerve and retina.

Reduced central corneal thickness with deep stromal scar on the right eye and the acute corneal edema suggested the diagnosis of keratoconus on both eyes, with acute keratoconus of the left eye. In addition, a connective tissue disorder causative for the blue discolored sclera was suspected.

Molecular analysis of the genes* ZNF469* and* PRDM5* was performed by polymerase chain reaction amplification and direct DNA sequencing. No mutation was found in ZNF469, but the homozygous variant c.17T>G, p.V6G, was detected in* PRDM5*. To exclude a dosage effect, a quantitative real-time PCR analysis of* PRDM5* showed no larger deletion or duplication. This variant c.17T>G, p.V6G, was not found in the 2504 control subjects in the “1000 genomes project” [8]. It does not affect any known functional domain but was predicted to be “disease causing” by MutationTaster. Based upon this prediction, the heterozygous state in the parents, and the absence in control populations the variant was thought to be likely disease causing.

**Figure 1:**
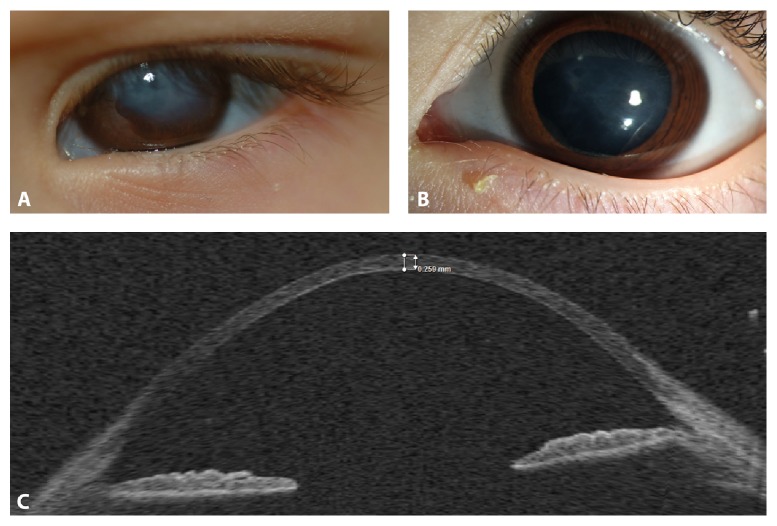
Acute keratoconus with hydrops in a 3-year-old child with brittle cornea syndrome. (A) Left eye three months after acute keratoconus with already clearing corneal cloudiness. (B) Seven months after acute keratoconus only little subepithelial scars remained. (C) Optical coherence tomography (OCT) at 7 months after acute hydrops demonstrated steepness and thinning of cornea. Corneal thickness centrally was 259 *μ*m.

To avoid amblyopia, there was an occlusion performed of the right eye for two hours per day and glasses were prescribed. Seven months after acute keratoconus, corneal cloudiness was nearly completely cleared under local antibiotic and hyperosmolar treatment with only little subepithelial scars remaining (Figures 1(A) and 1(B)) and a visual acuity of 0.2 in the left eye. Optical coherence tomography (OCT) showed steepness of the cornea and a central pachymetry on the left side of now 259 *μ*m (Figure 1(C)). A clear fundus view was given, so that at this moment there is no keratoplasty indicated.

**Figure 2:**
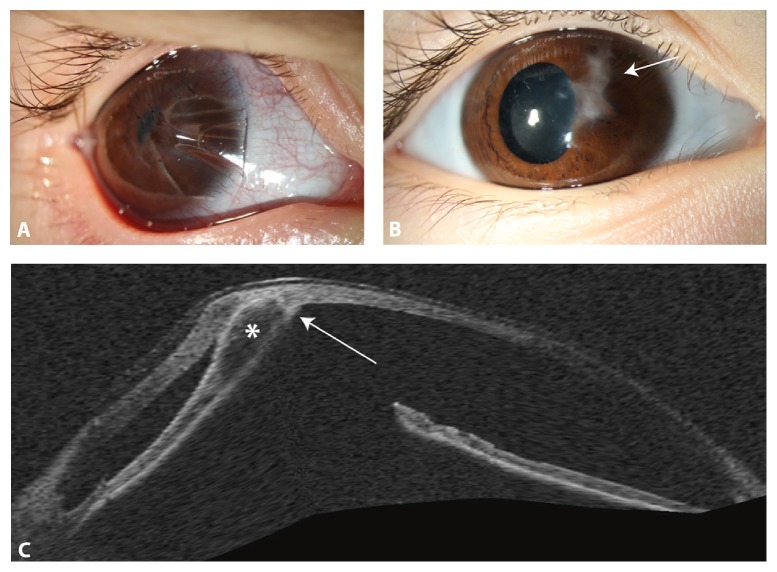
Spontaneous corneal perforation in a child with brittle cornea syndrome. (A) For primary repair of the spontaneous corneal rupture of the right eye 4 corneal sutures were placed and the right eye was covered with a contact lens. (B) After suture removal, cornea healed and mild iris incarceration (arrow) persisted. (C) Optical coherence tomography (OCT) showed the iris incarceration (*asterisk∗*) to be only adherent on the rear surface of the cornea (arrow). Corneal thickness was 289 *μ*m.

**Figure 3:**
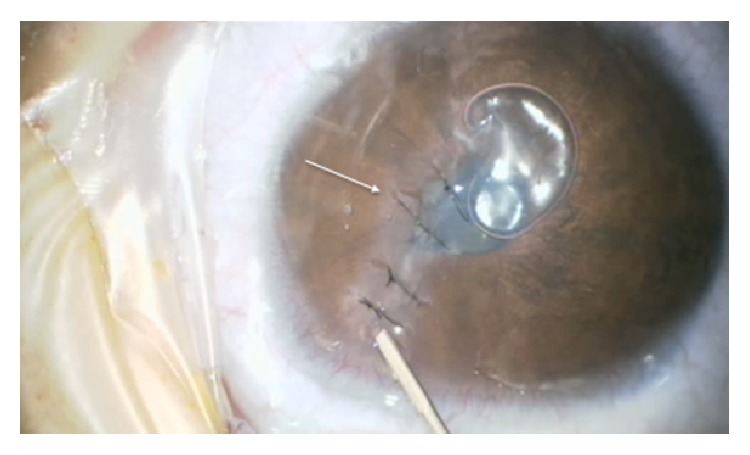
Intraoperative picture during surgical repair of spontaneous corneal rupture in brittle cornea. Note obvious cheese wiring (arrows) and attempt to maintain anterior chamber by intracameral air injection.

Five months after first consultation and while the left cloudiness was getting better, the 3-year-old boy presented with a large spontaneous corneal perforation on the right side. The spontaneous perforation extended from the pupil area to the peripheral 1 o'clock position with peripheral iris incarceration. The anterior chamber was flattened and fibrin was seen in front of the lens. After trying to reposition the iris incarceration, 4 corneal sutures were performed. Immediately there was cheese wiring (Figure 3) because of the extremely thin corneas of about 150–200 *μ*m. No more sutures could be fixed. Because of the extreme corneal thinning, there was no opportunity to perform an amnion-transplantation or keratoplasty. Spontaneous wound healing was observed using a bandage contact lens, systemic carboanhydrase inhibitor therapy, and ocular compression bandage for 7 days (Figure 2(A)). The examination after 4 weeks showed a complete corneal epithelialization with iris incarceration but deep anterior chamber and negative Seidel test. Two months after spontaneous corneal rupture another examination under general anesthesia was performed. All 4 sutures were loose so that all of them had to be removed. The pupil seemed rarely round, the optical axis was clear (Figure 2(B)), and fundus evaluation showed normal results. Using OCT, the iris incarceration was seen to be only adherent on the rear surface of the cornea (Figure 2(C)). Six months after spontaneous rupture, iris incarceration was reduced so that optical axis was free and no more surgical intervention was necessary. Best corrected visual acuity (BCVA) of the right eye was 20/400.

## 3. Discussion

Brittle cornea syndrome is a generalized connective tissue disorder associated with* ZNF469 *and* PRDM5* gene mutations [1, 3]. Not only ophthalmologic but also systemic findings reduce quality of life separated into auditory, skin, and musculoskeletal disorders (Table 1).

For example, progressive deafness, especially for higher frequencies, is reported. Deafness is caused either by conductive, sensorineural, or mixed disorders. In addition tympanic membrane can be hypercompliant [3]. Skin is pasty and soft with mild hyperelasticity. In childhood most patients suffer from musculoskeletal hypotonia and hip dysplasia. Pes planus, hallux valgus, mild contractures of the fingers, and arachnodactyly can occur [3].

Molecular analysis of our patient identified a novel homozygous variant in the* PRDM5 *gene c.17T>G, p.V6G. This homozygous variant has not yet been described to our knowledge. According to the MutationTaster, this mutation was estimated as pathogenic disease causing brittle cornea syndrome. Molecular analysis of the parents showed the same mutation in p.V6G in exon 1 of* PRDM5* gene but as a heterozygous status with the absence of ophthalmological findings. Unfortunately it is not possible to rule out the occurrence of this mutation in a Turkish cohort.

Other typical mutations in brittle cornea syndrome type 2 are located on chromosome 4 on the* PRDM5* gene, with cytogenetic location 4q27 or c.1768C>T [6, 9, 10]. The* ZNF469* gene mutations at chromosome 14 are responsible for brittle cornea syndrome type 1. Mutations of exons 9 till 14 are often found [6]; in addition mutations with cytogenetic location 16q24.2 are reported [11].

For the correct diagnosis of brittle cornea syndrome, molecular testing is essential. Differential diagnoses of brittle cornea syndrome also presenting with corneal thinning and blue sclera include an atypical congenital hereditary endothelial dystrophy 2 (CHED), Ehlers Danlos syndrome especially type VI, and osteogenesis imperfecta [2, 3, 12].

Also genetic testing in family members should be performed, especially to detect heterozygous mutation carriers to calculate the risk for further children [3]. Audiometry and tympanography are needed as well as orthopaedic check-ups and echocardiography to detect cardial failure [3]. Ophthalmologic consultations are necessary to monitor corneal thickness and to undertake preventive actions avoiding corneal injury. According to Amsler-Krumeich classification of keratoconus (Table 2) the progression of corneal thinning and the severity of keratoconus can be graded in four stages [13, 14]. This is mandatory since progress of corneal thinning increases the risk of spontaneous corneal or sclera rupture. Almost all patients loose visual acuity from complications of corneal rupture and scars [3]. The first step to protect the ocular surface is to prescribe protective polycarbonate goggles, so that scratching the eye is not possible anymore. Because of the young age of patients it is often necessary to train lifestyle behavior to reduce eye-hand contact and eye rubbing, but also parents and other caregivers must be introduced to minimize risks of ocular rupture [2, 3]. Contact sport activities should be avoided. Besides that correction of the visual acuity caused by myopia, keratoconus, and irregular astigmatism is necessary. Contact lenses are not indicated because of the corneal thinning and trauma risk. There were some trials with modified collagen cross-linking to stabilize corneal integrity, and first results showed visual improvement [3]. In cases of corneal rupture primary repair is required. Keratoplasty in childhood is difficult in general because of the high risk of immune responses [15–17]. Additionally in individuals suffering from brittle cornea syndrome, corneal thickness is extremely reduced and complications due to difficulties in corneal suturing increase [2, 3, 18, 19]. Also sometimes limbus-to-limbus transplantation technique becomes necessary to allow for scleral sutures [20].

Surgical repair of spontaneous corneal ruptures in brittle cornea syndrome is difficult. Here we achieved corneal sealing by a combination of cheese wiring sutures with bandage contact lens, pressure patches for one week, and systemic carboanhydrase inhibitors. Alternatively repair of corneal perforations has been described using tissue adhesives in dry conditions, viscoelastic agents, onlay epikeratoplasty, and gas/air tamponade [21]. Intraoperative optical coherence tomography can be of great value to examine these patients with reduced visibility of anterior segment structures [22–25].

Early diagnosis of brittle cornea syndrome is the most important step in the treatment of individuals suffering from this syndrome. Early treatment with protective glasses and observing the rules of conduct can prevent corneal rupture and so minimizes the risk for vision loss. Ophthalmic long-term follow-up and general follow-up examinations are needed to prevent systemic, extraocular disorders by this multisystemic connective tissue disorder.

## Figures and Tables

**Table 1:** Typical connective tissue disorders with ocular, auditory, musculoskeletal and cutaneous features in brittle cornea syndrome (modified from [1]).

Ocular features	
Keratoconus	
High myopia	
Progressive central corneal thinning	
Retinal detachment	
Irregular astigmatism	
Corneal or scleral spontaneous rupture	
Vision loss	
Auditory features	
Progressive deafness for higher frequences	
Hypercompliant tympanic membrane	
Cutaneous features	
Mild hyperelasticity	
Soft and pasty skin	
Musculoskeletal features	
Pes planus, Hallux valgus	
Hip dysplasia	
Contracture of the fingers or arachnodactyly	
Musculoskeletal hypotonia	

**Table 2:** Amsler-Krumeich classification of keratoconus (modified from [1, 2]).

Classification	Ophthalmological findings
Stage I	Myopia and/or astigmatism <5.0 D
	Mean central corneal readings <48.0 D
	Eccentric corneal steepening

Stage II	Myopia and/or astigmatism 5.0–8.0 D
	Mean central corneal readings <53.0 D
	Absence of scarring

Stage III	Myopia and/or astigmatism 8.0–10.0 D
	Mean central corneal readings >53.0 D
	Absence of scarring

Stage IV	Central corneal scarring
	Minimal corneal thickness 200 *μ*m
	Mean central corneal readings >55.0 D
	Refraction not measurable
